# A review of the mosquito-borne flaviviruses: Dengue virus and West Nile virus in Southern Africa

**DOI:** 10.1007/s13337-025-00917-x

**Published:** 2025-04-10

**Authors:** Maropeng C. Monyama, Letlhogonolo R. Molefe, Stephen Meddows-Taylor

**Affiliations:** Department of Life and Consumer Sciences, University of South Africa, Private Bag X6, Florida, Johannesburg 1710 South Africa

**Keywords:** Southern Africa, Dengue virus, West Nile virus, Mosquito-borne, Flavivirus

## Abstract

Dengue virus (DENV) and West Nile (WNV) viruses are important re-emerging mosquito-borne members of the genus *Flavivirus* that are under-recognized in many parts of Africa. This review aims to evaluate the existing literature on the transmission, epidemiology, diagnostic techniques, clinical presentation and prevention of infection with DENV and WNV in Southern Africa. Literature shows that both DENV and WNV are transmitted by mosquitoes of *Aedes spp.* and *Culex* species., respectively, and both viruses are widespread in the Southern African region. Epidemiologically, sporadic outbreaks have been reported of both DENV and WNV in various Southern African countries, indicating the ongoing threat of these viruses. However, the lack of comprehensive surveillance and diagnostic capacity challenges accurate estimation of their true prevalence. Diagnostic techniques for DENV and WNV involve serological tests, molecular tests and viral isolation, enabling prompt diagnosis and differentiation from other febrile illnesses. In Southern Africa, infection with DENV and WNV presents significant public health concerns, with the clinical presentation of both infections ranging from asymptomatic cases to severe manifestations. Symptoms of infection include high fever, myalgia, rash, and, in severe cases, haemorrhagic fever for DENV and neurological complications for WNV. No specific antiviral treatment exists for either virus, underscoring the importance of supportive care and symptom management. To prevent the spread of DENV and WNV in Southern African countries, a combination of prevention and treatment strategies should be employed, including effective mosquito control, continuous monitoring of vector population dynamics, public health education, and surveillance and reporting systems for averting future outbreaks.

## Introduction

The genus *Flavivirus* includes arthropod-borne viruses that are transmitted to vertebrates by infected ticks and mosquitoes, producing disease in human beings and animals [22]. Most Flaviviruses are transmitted through the bite of an infected mosquito, mostly by the *Aedes* genus including *Aedes aegypti* and *Aedes albopictus*, and *Culex* mosquitos [7, 12]. Transmission by mosquitoes to people has been reported for Dengue virus (DENV), and West Nile virus (WNV) among others, although human beings are usually dead-end hosts, since viremia levels are not thought to be high enough to infect additional mosquitoes [22]. Consequently, these arthropod-borne viruses are associated with emerging and re-emerging human diseases, including dengue haemorrhagic fever, Kyasanur Forest haemorrhagic disease, Japanese encephalitic disease, Rocio virus encephalitis and West Nile fever [21].

DENV and WNV are important re-emerging mosquito-borne members of the genus *Flavivirus* that are under-recognized in many parts of Africa due to a lack of surveillance [6, 7, 55, 72]. In African countries that lack routine arboviral surveillance programs, the transmission and overall burden of DENV and WNV infection is largely unknown, and without such information, these countries cannot monitor, detect and respond to outbreaks adequately [72, 73]. Both DENV and WNV flaviviruses have a single-stranded positive-polarity RNA genome of approximately 11 kb [12, 19, 55,]. DENV and WNV can cause significant morbidity and mortality, making them a major public health concern in many regions around the world, including the Southern African region [7].

**Fig. 1:**
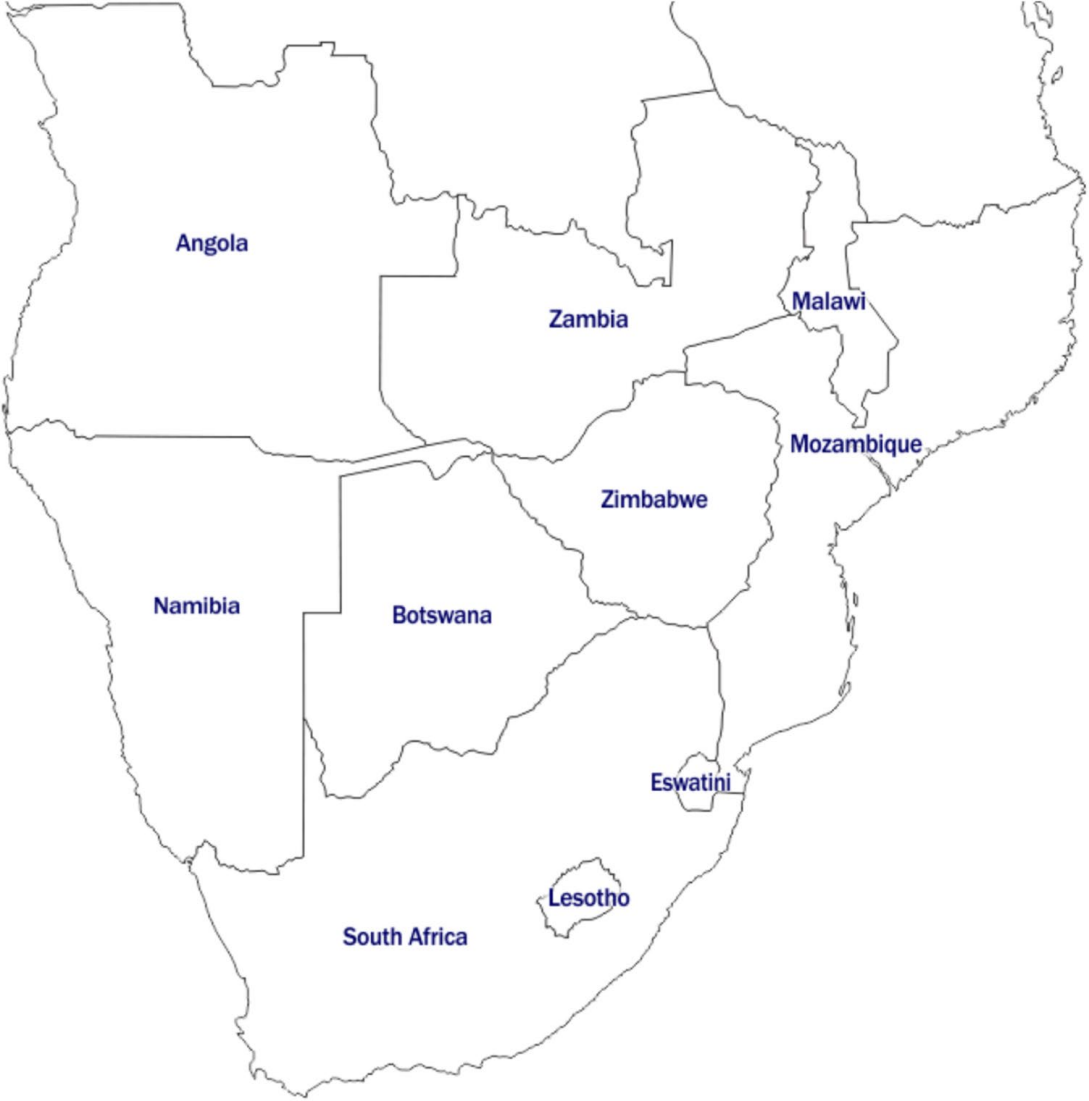
The Southern African region with individual countries indicated (modified from https://d-maps.com/carte.php?num_car=4359&lang=en)

Climate change and the increased movement of both humans and animals have facilitated the spread of these viruses to new areas, leading to outbreaks and epidemics in previously unaffected regions. Human infections with WNV tend to be sporadic and large epidemics occur when unusually high rainfall or hot weather favours the breeding of mosquito vectors [11, 80]. Although sporadic dengue fever has been observed for more than 200 years, the reasons for the global resurgence of epidemics of dengue fever and dengue haemorrhagic fever are not very clear [4, 39]. In southern Africa, WNV was found to be widely endemic in areas where the principal vector, *Culex univittatus*, and avian hosts of the virus are present [11, 80], whilst DENV was found in areas where principal vectors of *Aedes* genus are present [5, 59]. This article, therefore, aims to review the existing literature on the epidemiology, diagnostics, preventative measures, and factors that lead to the outbreak of DENV and WNV in the Southern African region. The Southern African region, which includes the countries of Angola, Botswana, Lesotho, Malawi, Mozambique, Namibia, South Africa, Eswatini, Zambia, and Zimbabwe, encompasses the southernmost area of the African continent, as shown below in Fig. 1.

### Epidemiology of WNV

WNV was first discovered in the West Nile district of Uganda in 1937 in a febrile human patient [51, 63, 65]. Since then, sporadic outbreaks have been reported from the 1950s through the 1980s when the virus was described in other Southern African countries (Table 1) including South Africa [12]. In South Africa, the seropositivity of WNV was demonstrated in blood samples collected in the year 1954 from individuals who had never travelled out of the country [12].

**Table 1:** Summary of various WNV outbreaks in South Africa

Area of outbreaks		Year	Cases	Serotype	References
South Africa	Karoo	1974	6	–	[43]
	Witwatersrand-Pretoria	1983–1984	5	–	[31]
	Karoo	2010	21	–	[52]
	Highveld				
	KwaZulu-Natal				
	Pretoria	2008–2009	36	–	[81]

Five genetic lineages of WNV exist worldwide, however, the two major lineages from a public health perspective are WNV-Lineage 1 and WNV-Lineage 2. This is because these lineages are implicated in several epidemics in various countries, and they are most pathogenic and widespread. These two lineages are under-reported in African countries [44, 65, 71] and were identified after the discovery of WNV in Uganda,thus, WNV-Lineage 2 has been reported to be endemic in the Southern Africa region [65]. In Southern Africa regions, WNV-Lineage 2 circulation has been reported in countries like Botswana, Mozambique, Namibia, and South Africa [18, 44]. In Zambia, the WNV isolate was closely related genetically to WNVL-2 South African strains, which have been previously shown to be highly neuroinvasive [56].

Historically, the WNV-lineage 2 was considered for a long time to be less pathogenic than WNV Lineage 1, until its evolution became more virulent and caused severe disease forms in South Africa other than among humans and birds in Europe [14], Fall et al. 2017; [44]. The WNV-lineage 2 strains have been identified in humans and horses with febrile and neurologic disease in southern Africa and Madagascar [27, 70]. Although WNV-lineage 2 is predominantly associated with neurological infections of WNV in humans and animals in South Africa, a few WNV-lineage 1 strains have also been identified, suggesting that migratory birds may also import these strains to the region [8]. The WNV-lineage 2 strain reported in Zambia was shown to be closely related to strains circulating in South Africa, which have been reported to be associated with neurological disease in humans [49]. Also, genetic characterization studies have revealed the presence of WNV lineages 1 and 2 in Zambia [56, 62].

Human cases of WNV fever have been diagnosed in South Africa and the detection of antibodies against WNV in humans has been documented in both South Africa and Zambia [51, 8]. WNV fever cases in humans have been consistently diagnosed in South Africa, with a substantial outbreak in the Karoo in 1974 [8], followed by an outbreak that occurred in Gauteng province in the year 1984. This was the result of unusually high rainfall and flooding in the area [29]. The National Institute for Communicable Diseases (NICD) in South Africa, reports approximately 5–15 cases of WNV fever annually [11, 65].

WNV was detected in 3.5% of mysterious cases of human neurological disease in Gauteng provincial hospitals in South Africa from 2008 to 2009, which indicates that WNV is mostly underdiagnosed in human neurological cases [10, 81]. A study conducted by van Eeden et al., [15] in South Africa between 2011 and 2012 on antibodies against West Nile and Shuni viruses in veterinarians, demonstrated a 7.9% WNV seroprevalence among veterinarians. In Zambia, a study by Mweene-Ndumba and colleagues was the first to document the seroprevalence of WNV infection and its correlates [51]. The results of the survey revealed a seroprevalence of 10.3% (370/3625) for WNV among participants in the North-Western and Western provinces in the country. This is the only study and report of WNV in humans in this country. Additional studies on WNV in Zambia were in *Culex* mosquitoes at an occurrence of 6.7% and in farmed crocodiles [56]

### Vector transmission of WNV

**Fig. 2:**
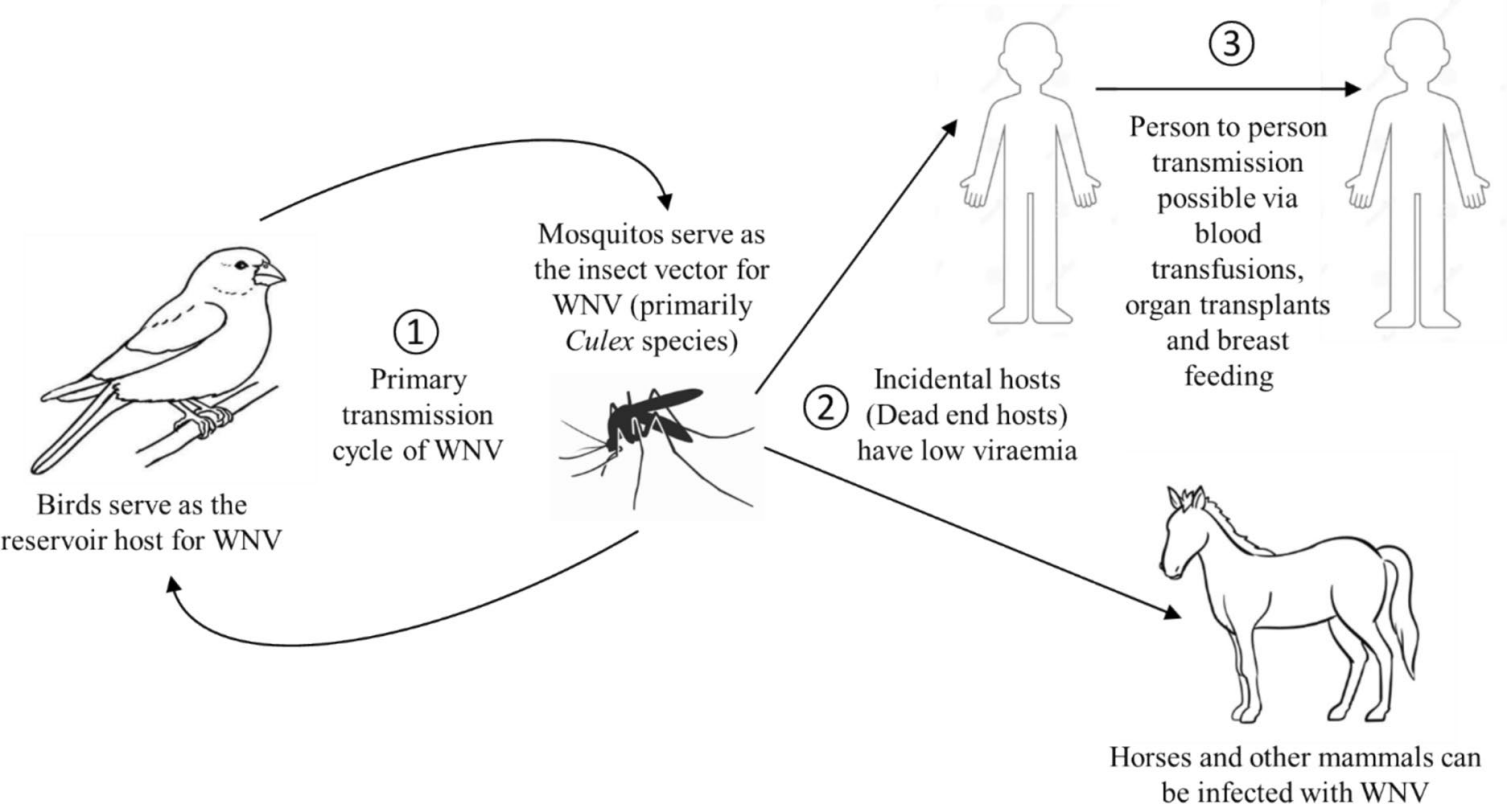
Transmission cycle of WNV. Mosquitos with WNV bite and infect people, horses, and other mammals

WNV is a zoonotic mosquito-borne *Flavivirus* maintained in a bird-mosquito-bird cycle. The virus is incidentally transmitted to humans and other mammals, which are dead-end hosts, by the bite of infected mosquitoes *Culex sp* [20]. The virus is primarily spread when a mosquito is exposed to the virus by feeding on birds that develop high levels of the virus in the blood [Fig. 2] [65]. Migration is often cited as the route for the virus to be transported from southern Africa to Europe and beyond as birds are the natural host and they play a major role in the geographic dispersion of the virus [51, 65]. Humans become infected by the virus through mosquito bites [51, 68]. The abundance and feeding patterns of infected mosquitoes, as well as human behaviour that influence their exposure to mosquitoes, influence the likelihood of WNV transmission [65]. Person-to-person transmission of either virus by *Culex univirratus* can be discounted because of the extremely low titres that are reached in the human peripheral bloodstream [31, 43].

Though WNV is mostly transmitted to humans by *Culex* mosquito species, *Aedes spp* mosquitoes may also serve as an important transmission vector of WNV [12, 51]. In the year 1950, experimental transmission was reported in *Culex pipiens* and *Culex tritaeniorhynchus*. These two species are found across large areas of the African continent [65]. However, In South Africa, *Culex univittatus* has been implicated as the primary WNV vector based on field isolation rates. Other WNV isolates were also obtained from *Culex theileri*, *Culex pipiens*, *Culex neavei*, *Aedes caballus*, *Aedes circumluteolus* and *Coquillettidia* spp. [44]. Mosquito surveillance studies conducted in Zambia have identified WNV isolates from *Culex quinquefasciatus* mosquitoes collected in the Western Province of the country [56].

### Clinical symptoms and pathogenesis of WNV

While severe cases may be biphasic and exhibit symptoms for as long as 60 days, most infections are typically asymptomatic and appear 3–14 days after a mosquito bite carrying WNV. They can also last for an additional 3–6 days [12, 65]. Symptomatic infections may vary from flu-like sickness to serious neuroinvasive diseases, for which there is no specific treatment [12]. Other symptoms include headaches, rash, nausea, vomiting, diarrhoea, conjunctivitis, abdominal pain, pancreatitis, myocarditis, hepatosplenomegaly, muscle and joint pains or hepatitis [11, 26, 51, 65]. Benign West Nile fever in humans is a febrile illness, with myalgia, arthralgia, lymphadenopathy, and often maculopapular or roseolar rash [11]. Few patients have serious complications such as acute aseptic meningitis, encephalitis, necrotic hepatitis, severe headache, flaccid paralysis and occasionally death [11, 65].

### Vector control, prevention, laboratory diagnosis and treatment of WNV

A survey by MacIntyre and colleagues [36] of WNV and Banzi Viruses in mosquitoes has shown that there is a wide range of new potential vectors for arboviruses that require further investigation. For example, WNV was identified in mosquitoes in areas at the animal or human interface in South Africa. This suggests an increase in the circulation of potential viruses including WNV between humans, wildlife, domestic animals, and avian species that are common in those areas. A study by Mweene-Ndumba and colleagues [51] in Zambia suggested that surveillance at the border between Zambia and Angola should be enhanced to at least reduce the importation of WNV into Zambia. Regarding vector management and control, the study has shown that participants who lived in a house sprayed with residual insecticide were less likely to be infected with the virus compared to those who did not have their houses sprayed. Thus, for one to minimize the contact between mosquito to man, the use of insecticide residual spray is highly recommended [51]. Prevention is essential in avoiding WNV infection and using measures such as mosquito repellents, insecticide-treated bed nets, wearing protective clothing such as long-sleeved shirts and long pants, eliminating standing water that serves as mosquito breeding grounds, and installing screens on windows and doors can help reduce the risk of infection [33].

Diagnosing WNV infection involves a combination of clinical evaluation, laboratory testing, and epidemiological information. Since symptoms of WNV infection can overlap with other diseases such as bacterial meningitis, influenza, or other viral encephalitis’s, a differential diagnosis is essential to rule out other potential causes of infection [67]. The most used laboratory method for WNV infection is to detect WNV-specific IgM antibodies by ELISA in either cerebrospinal fluid or serum [67]. The detection of viral RNA in blood or cerebrospinal fluid by RT-PCR can be particularly useful for confirming acute infections, especially in cases of neurological involvement. Detection of IgM in serum or cerebrospinal fluid (CSF is the preferred method for diagnosing WNV infection,however, because of cross-reactivity between flaviviruses, positive results should be confirmed by virus neutralization assay [81]. Early WNV infection can be diagnosed by PCR and virus isolation [60], but success has been limited in diagnosing more advanced diseases with these techniques [81]. The identification of WNV in mosquitoes found in the Southern African regions, confirms that the WNV could be circulating within the communities, and this suggests the need for an appropriate understanding of disease epidemiology in those communities [69].

Currently, six effective licensed vaccines, either attenuated, as against Yellow fever, Dengue and Japanese encephalitis viruses, or inactivated against Japanese encephalitis, tick-borne encephalitis and Kyasanur forest disease viruses, are available. However, none of the vaccines tested in humans for WNV have progressed further than phase I/II clinical trials and none has been licensed for human use against WNV [32]. Nevertheless, preventive measures against infection with WNV are encouraged [51]. Thus, there is no specific prophylaxis or treatment that exists against the WNV disease in humans [20]. Although most individuals infected with WNV experience mild symptoms and recover without specific medical treatment, severe cases of WNV infection do occur, where medical care may be necessary to manage complications and provide supportive treatment [33].

Severe cases of WNV may require hospitalization for close monitoring and treatment of complications such as encephalitis or meningitis, and additional treatment in severe cases may include intravenous fluids, pain management to reduce fever and alleviate body aches, and mechanical ventilation or oxygen therapy in severe cases that affect the respiratory system [35]. Though there is currently no specific antiviral treatment or cure for WNV infection, interferon α-2b may reduce disease severity and complications, even if administered after several weeks of WNV infection [35]. In vitro studies on ribavirin for WNV have shown activity against the virus, although only at very high doses [28, 47]. Additionally, ribavirin administered orally to patients infected with WNV in an outbreak in Israel did not appear to have any beneficial effect on patient outcomes [13].

### Epidemiology of DENV

Dengue virus (DENV) is a subject of global public health importance, a mosquito-borne viral disease with extensive influence on human health especially in low-resource countries [19, 23]. This virus is believed to be the most significant arbovirus based on its rapid spread combined with its high burden of morbidity and mortality [55, 59]. Accordingly, the virus is transmitted to susceptible vertebrate hosts by hematophagous mosquitoes [69].

There has been fragmented reporting on the epidemiology of DENV on the African continent [19]. In Southern African regions, the epidemiology and public health impact of DENV is not reported, but DENV is mostly endemic in parts of Southern Africa with favourable conditions for vector increase, disease transmission, and spread [59]. The incidence and distribution of mosquito-borne arboviruses including DENV, characterize a serious threat to global health [2]. The occurrence of DENV cases in the regions is certainly under-described, which is due to the following: (i) low awareness of the medical community or healthcare providers, (ii) lack of laboratory testing, and (iii) poor or non-existent surveillance programs [2].

DENV cases were first recorded in Africa in the year 1779 and there have been at least three events of endemic transmission of DENV in South Africa, following importation through infected human travellers [23]. These caused outbreaks in KwaZulu-Natal in 1897, 1901 and in the summer of 1926–1927. During this outbreak, 50,000 cases and 60 deaths were reported [19, 23, 25, 48]. This epidemic in South Africa was confirmed by retrospective neutralizing antibody testing in the mid-1950s, however, the other reported epidemics were not laboratory-confirmed and consequently may not have been DENV [6].

Notably, DENV is endemic in Mozambique and at the formal and informal Mozambique-South Africa border crossings [48]. Since the 1926–1927 outbreak, there has been a series of outbreaks of all four DENV serotypes (namely DENV-1, DENV-2, DENV-3, and DENV-4) across the African continent [19, 23, 25]. Most DENV-detected cases in Angola occurred in urban settings, and the peak of confirmed cases between April and May 2018 occurred in months of high mosquito abundance. Nevertheless, confirmed cases were detected throughout the year, suggesting that some part of the region’s climate provides year-round opportunities for the transmission of *Aedes aegypti*-borne viruses [54].

Of the four known DENV serotypes, DENV-1, DENV-2, and DENV-4 have been reported as circulating serotypes in Southern African countries including South Africa, Mozambique, and Angola [1, 30, 55]. Before the 2014 outbreak, DENV-1 was identified during the previously reported outbreaks in southern Africa [1]. However, in Mozambique, the last known outbreak of DENV that occurred in 1984–1985 in northern Mozambique was identified to be caused by DENV-3 [40]. In 1985, individuals who returned to Durban after visiting India suffered DENV-1 infection, and between 1997 and 2003, there were eleven more imported cases of which two have been typed as DENV-1 and DENV-3 strains [30].

The imported DENV cases identified in Gauteng, Western Cape, and KwaZulu-Natal had a prevalence of 48%, 28% and 19% respectively [48]. Dissemination of DENV-1 in Angola was detected in 2013, while DENV-type 2 was detected in 2014 and 2018 in Mozambique and Angola, respectively [54, 40]. Evidence for the existence of DENV in Zambia was initially shown serologically in the year 1987. Since then, serological evidence of the virus has been reported in the Western, North-Western and Central Provinces of the country with a reported prevalence ranging from 4.1 to 16.8%. [6, 42, 69]. Evidence of the circulation of DENV serotype-2 was primarily described in Zambia in 2014 [42].

A study from Mozambique by Ali et al. [5] has shown that IgM antibodies against DENV have been identified in 7.1% of participating individuals. These results are slightly lower than those reported in a previous outbreak of DENV in the Cabo Delgado province in Northern Mozambique [55]. This suggests a well-established endemic transmission of DENV in Mozambique, with periodic epidemic transmission that facilitates the emergence of outbreaks as shown in other countries in the continent [45, 46]. A study by Mazaba-Liwewe and colleagues [42] reported DENV IgG seroprevalence as 4.1% in the Western and North-western regions of Zambia. The study by Parreira et al. [59] has demonstrated that DENV has been repeatedly found in travellers returning from countries that border Angola including the Democratic Republic of Congo and Namibia [54]. Also, from 2000 to 2016, returned travellers who were managed by South African healthcare facilities for fever of unknown origin were referred for Dengue diagnostic testing at the South African NICD, and 176 cases of DENV were confirmed by laboratory diagnostics [48]. DENV outbreaks are commonly observed in regions characterized by rapid and unexpected urbanization, alongside intense migration [40]. These outbreaks have been reported in some parts of Southern Africa (Table 2), including Angola, Mozambique, and South Africa. The most recent outbreak in Mozambique occurred in 2014, with over 400 cases reported [40].

**Table 2:** Summary of various DENV outbreaks across various Southern African countries

Area of outbreaks		Year	Cases	Serotype	References
South Africa	KwaZulu-Natal	1897, 1901	50000	–	[48]
		1926–1927	–	–	[48]
					[34]
	–	2000–2016	176	–	[48]
	Durban	1997–2003	> 11	DENV-1 and DENV-3	[30]
		2014	–	DENV-2	
Angola		1985	–	DENV-1	[54]
		2013	–	DENV-1	
		2018	–	DENV-2	
Mozambique		1984–1985	–	DENV-3	[40]
		2014	> 400	DENV-2	

A range of factors that have influenced the spread of this virus in the regions include high temperatures, heavy rainfall, inadequate waste disposal management, unintended urbanization, high population density, and global travel [55, 59]. In addition, substandard housing, high air humidity, and poor sanitation also provide suitable environmental and microecological conditions for *Aedes aegypti* survival and proliferation in urban and semi-urban areas [40]. These features are commonly seen in some of the Southern African regions, particularly in Mozambique. Hence changes in climate, population, plastic pollution, and tyre build-up are anticipated to worsen the DENV dissemination in the regions [19].

### Vector transmission of DENV

**Fig. 3:**
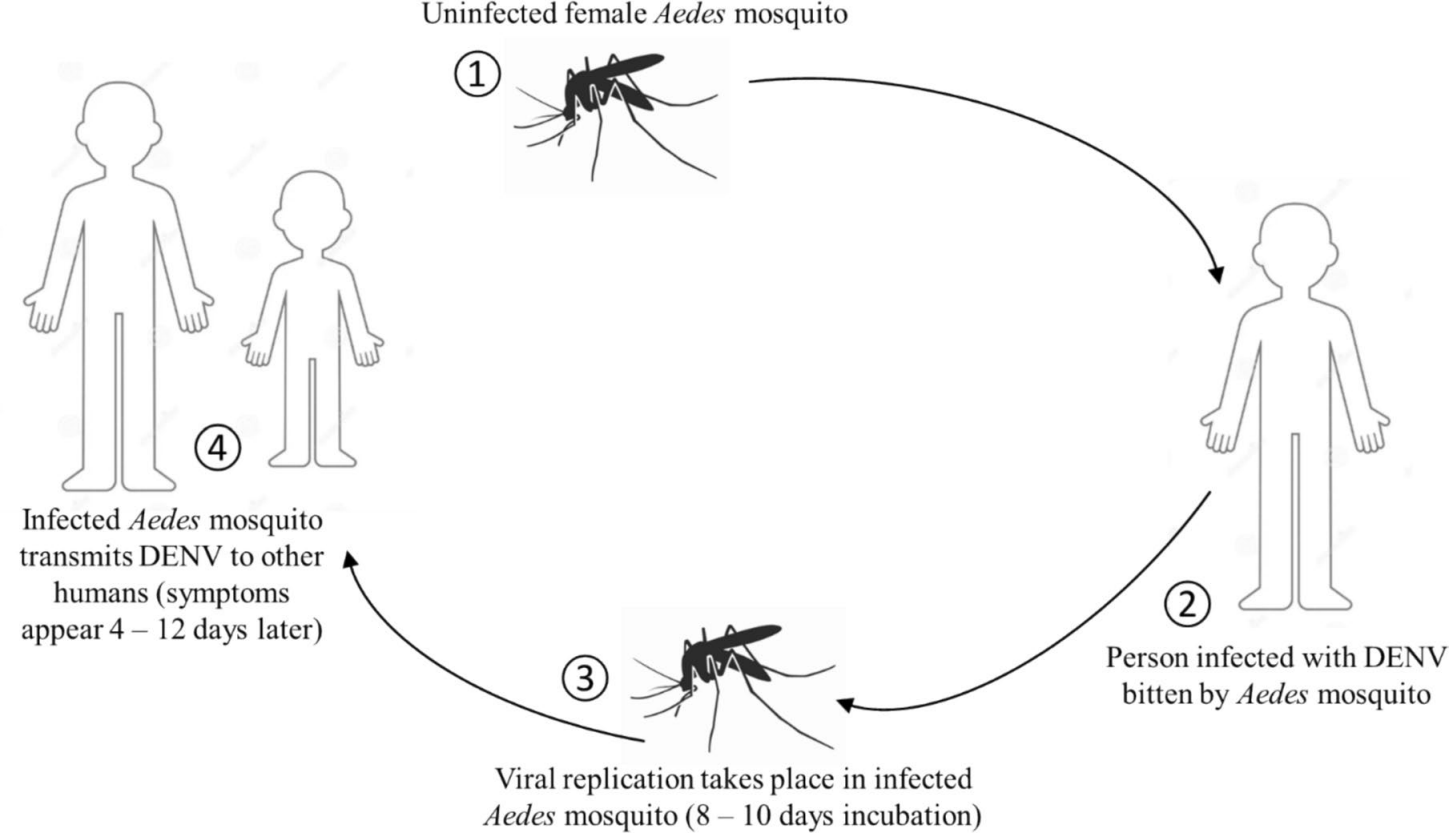
A cycle illustrating how DENV is transmitted from *Aedes aegypti* mosquitos to humans

The primary vector of the DENV is the anthropophilic *Aedes aegypti* mosquito, although transmission by the *Aedes albopictus* mosquito has also been shown. Transmission of all four DENV serotypes is maintained by horizontal transfer in an *Aedes aegypti*-human cycle as shown in Fig. 3. Female mosquitoes consume viraemic blood from a human host to become infected. Vectors become infectious and can spread viruses by biting after surviving an extrinsic incubation period of 8–10 days. The length of the extrinsic incubation is inversely dependent to ambient temperature within high and low extremes, at which vectors perish or viral replication stops. Virus incubation in humans lasts for 4–12 days (usually 4–7 days) [61, 75].

*Aedes* mosquito vectors are abundant in many geographical areas in Southern African countries including Botswana, Mozambique, and Zimbabwe [59]. The vector of dengue fever *Aedes aegypti* is present in certain areas of South Africa, including the KwaZulu-Natal coastline [53]. The distribution of *Aedes aegypti* is elevated along the Indian Ocean coastline and in the Eastern portions of Limpopo and Mpumalanga provinces in South Africa. The population of this species is also found in the Gauteng Province and in Southern Limpopo [48]. The presence of major arbovirus vectors may signify that the transmission of certain arboviruses of public health importance may involve multiple-vector systems [59]. Thus, there is a need for a systematic understanding of the occurrence and arbovirus transmission role, of the overlooked potential vectors of public health importance in Southern African regions [2].

### Clinical symptoms and pathogenesis of DENV

The basic pathogenesis of DENV infection is similar regardless of the region where it occurs, and in Southern Africa, the pathogenesis of the virus is similar to other parts of the world where the virus is present. Additional research to understand the pathogenesis of DENV in Southern African regions is still required [9]. DENV has the potential to cause a range of disease symptoms from asymptomatic mild infections to severe, and even fatal manifestations [19]. The clinical presentation of this virus is non-specific and sometimes can lead to misdiagnosis since it has a similar clinical presentation to other arboviruses [69]. Clinical symptoms caused by DENV are characterised by fever, severe headache, retro-orbital pain, nausea, vomiting, myalgia, arthralgia, thrombocytopenia, rash, and mucosal bleeding from the gums, nose and the gastrointestinal system [54, 76]. Occasionally, infection causes a potentially lethal complication called severe DENV that is characterized by increased vascular permeability, which can lead to fluid accumulation, haemorrhage, and shock [54, 76].

The pathogenesis of severe dengue infection is thought to involve the activation of a complex immunological response that can cause damage to blood vessels and organs such as the liver and kidneys [3]. The manifestation of severe dengue is a sudden-onset shock syndrome called dengue shock syndrome, which is characterized by hypotension, plasma leakage, and haemorrhage [64]. Dengue haemorrhagic fever/dengue shock syndrome occurs mostly in individuals with secondary DENV infection with a different serotype and in infants with a primary infection born to dengue-immune mothers [24]. Other severe complications of DENV infection include encephalitis, myocarditis, and acute respiratory distress syndrome [66]. In a study by Parreira et al., [59] from Angola, fever was the most reported clinical sign, being frequently associated with myalgia and headache. This was done based on the complete notification epidemiological forms for DENV RT-PCR cases. Thus, the main clinical symptoms of the virus were fever, myalgias, headache and arthralgia with PCR-confirmed cases of 97%, 62%, 57.6% and 42% respectively. Consequently, some patients also reported retro-orbital pain (14%), and rash (4.5%) [54]. Notably, haemorrhagic signs correlated with plasma leakage were observed in 14% of the participating patients [54]. Acquiring any one of the DENV serotypes will naturally confer enduring immunity to the disease caused by that serotype. However, the immunity against that specific serotype does not cross-protect among other serotypes. Thus, individuals could be affected by DENV up to four times throughout their lifespan [19, 54]. Infection with a heterotypic serotype increases the risk of developing severe dengue disease [54].

### Vector control, prevention, laboratory diagnosis, and treatment of DENV

Detection of DENV cases on time can assist in the prevention of the outbreaks. Several Southern African countries are resource-constrained and consequently may encounter issues with appropriate diagnostic testing, vector control, and medical attention to DENV [19]. This will negatively affect the accurate tracking of the incidence and prevalence of the virus. Prevention and treatment strategies for DENV are crucial to controlling the spread of the disease in Southern African countries. DENV alone is estimated to cause 50–100 million clinical cases each year, nonetheless, the exact disease burden is unknown in most endemic countries [64, 78]. The main method of DENV control is to destroy vector mosquitoes and their breeding places.

Transgenic mosquitoes have been created by the British biotechnology company Oxitec, which had earlier completed a trial in the Cayman Islands in 2009–2010. The "sterile" male mosquitoes created by Oxitec convey a fatal genetic component to their progeny, rendering them unviable [57]. The Malaysian government also allowed the release of genetically altered infertile male A. *egypti* mosquitoes in November 2010 [16]. Since this is a new variation of the sterile insect technique, which is frequently employed in agriculture, with the exception that in this technique, insects are rendered sterile by radiation rather than genetic alteration, this self-limiting approach is viewed as being generally safe [16]. Another strategy being planned involves infecting *A. aegypti* mosquitoes with a strain of the symbiotic bacterium *Wolbachia*, which shortens their lifespan and hinders their capacity to transmit DENV to people [17]. If the experiment is successful, a biological control study will begin in Queensland, Australia, and then move on to Vietnam [17], [57]. Thus, effective mosquito control seems to be the most critical strategy to prevent DENV transmission, as the virus causes sickness for which there are no medications or treatments, and bed nets usually do not work as a barrier [58]. Hence, Southern African countries should prioritize efforts to eliminate mosquito breeding sites and use insecticides to kill adult mosquitoes.

Improved monitoring and disease surveillance are required to further assess DENV endemicity in the region [54]. In Mozambique, since the last outbreak of DENV, the Ministry of Health has made efforts to expand surveillance systems for acute febrile illnesses throughout the country [50]. In Angola, to improve the detection of arbovirus outbreaks, the National Arbovirus Laboratory of Surveillance Programme was set up at the National Institute of Health Research by the Ministry of Health, which formally kick-started arbovirus surveillance activities in early 2017 [54]. The South African government has implemented measures to prevent the spread of Dengue and other mosquito-borne diseases, including public education campaigns and vector control programs. In Botswana and Lesotho, vector control programs are also in place to prevent the spread of mosquito-borne diseases, although they are primarily focused on malaria control [48, 38].

Dengue misdiagnosis is also a problem in southern African regions, particularly due to the typical non-specific clinical presentation of DENV leading to misdiagnosis as malaria [19]. Lack of routine laboratory diagnostic capacity and clinical awareness of DENV delay the identification of the outbreaks, especially in a region where malaria is highly endemic [40].

Treatment of DENV includes supportive care, focusing on maintaining fluid balance, managing symptoms such as fever, pain, and headaches, also avoiding further infection [74]. In severe cases, hospitalization may be necessary, and intravenous fluids may be used to manage dehydration. Acetaminophen is recommended for pain relief, while aspirin should be avoided [79]. There are no specific antiviral therapies or vaccines available yet for DENV [37, 77]. In Southern African countries, the management of Dengue typically follows the guidelines of the World Health Organization, which emphasize early detection and treatment of the disease [74].

## Conclusion and recommendations

In Southern Africa, infection with DENV and WNV presents a significant public health concern. Prevention strategies for these two viruses focus on mosquito control measures, public awareness campaigns, and surveillance systems. Vector control is particularly vital in regions with susceptible ecological conditions. The continuous monitoring of vector population dynamics, temporal and spatial variation of mosquito breeding sites, and their environmental and bioecological associates, in addition to significant measures to efficiently regulate the *Aedes* mosquitos are recommended [40]. Human movement has been implicated in the spread and transmission of vector-borne diseases including DENV and WNV (Stoddard et al. 2009). It should therefore be emphasized that travellers take personal protective measures against mosquito bites as a preventive measure. As DENV and WNV continue to pose a substantial burden on Southern African healthcare systems, further research into their epidemiology, clinical management, and vaccine development remains imperative to mitigate their impact on public health in this region. Arboviruses are considered one of the most important health risks on the African continent due to its climate, and the various interactions between humans, animals and vectors [41]. The One Health approach, which integrates human, animal, and environmental health, offers a comprehensive strategy to prevent DENV and WNV infections in Southern Africa. Implementing surveillance and monitoring, vector control, community engagement and climate change adaptation, this approach could reduce the likelihood of outbreaks by addressing root causes in the environment and animal reservoirs to enhance preparedness and reduce the burden of these mosquito-borne diseases.
